# Venlafaxine ameliorates the depression-like behaviors and hippocampal S100B expression in a rat depression model

**DOI:** 10.1186/s12993-016-0116-x

**Published:** 2016-12-08

**Authors:** Chang-Hong Wang, Jing-Yang Gu, Xiao-Li Zhang, Jiao Dong, Jun Yang, Ying-Li Zhang, Qiu-Fen Ning, Xiao-Wen Shan, Yan Li

**Affiliations:** 1 Department of Psychiatry, The Second Affiliated Hospital of Xinxiang Medical University, Xinxiang, 453002 Henan China; 2 Standard Technological Co. Ltd. (Xinxiang Institute for New Medicine), Xinxiang, 453003 Henan China; 3 Xinjiang Hongda Food & Beverage Co. Ltd., Xinjiang, 043102 Shanxi China; 4 Department of Child and Adolescent, Public Health College, Zhengzhou University, 100 Kexue Road, Zhengzhou, 450001 Henan China

**Keywords:** Venlafaxine, Stress, Depression, Hippocampus, S100B protein

## Abstract

**Background:**

Accumulating evidence has indicated that S100B may be involved in the pathophysiology of depression. No published study has examined the effect of the antidepressant drug venlafaxine on S100B in animal models of depression. This study investigated S100B expression in the hippocampus and assessed the effect of venlafaxine on S100B mRNA level and protein expression in rats exposed to chronic unpredictable mild stress (CUMS).

**Methods:**

Forty Sprague-Dawley rats were randomly divided into four groups as control, 0, 5 and 10 mg venlafaxine groups. The venlafaxine groups were exposed to CUMS from day 2 to day 43. Venlafaxine 0, 5 and 10 mg/kg were then administered from day 23 to day 43. We performed behavioral assessments with weight change, open-field and sucrose preference, and analyzed S100B protein expression and mRNA level in the hippocampus.

**Results:**

The CUMS led to a decrease in body weight, locomotor activity and sucrose consumption, but venlafaxine treatment (10 mg) reversed these CUMS-induced decreases Also, CUMS increased S100B protein expression and mRNA level in the hippocampus, but venlafaxine treatment (10 mg) significantly decreased S100B protein expression and mRNA level, which were significantly lower than the other treatment groups, without significant difference between the 10 mg venlafaxine and the control groups.

**Conclusions:**

Our findings showed that venlafaxine treatment (10 mg) may improve the depression-like behaviors and decrease over-expression of S100B protein and mRNA in the hippocampus in a rat model of depression.

**Electronic supplementary material:**

The online version of this article (doi:10.1186/s12993-016-0116-x) contains supplementary material, which is available to authorized users.

## Background

Major depressive disorder (MDD) is one of the most common, serious mood disorders with a high recurrence rate, representing a major socio-economical burden [1]. However, the pathogenic mechanisms are still unclear. Understanding the causes and neurobiological basis of depression remains a challenge. Recently, it has been suggested that mood disorders are characterized by disease-specific glial pathology [2, 3]. Post mortem studies showed reductions in glial cell density or glial cell numbers in prefrontal brain regions in patients with mood disorders [4], mainly displaying alterations of astrocytes and oligodendrocytes [5].

S100B is a glia-derived neurotrophic marker and an acidic and calcium-binding protein that is primarily produced by astrocytes and oligodendrocytes in the human brain [6]. Astrocytes are the main type of glial cells and are distributed throughout the nervous system. They have a role in the nutrition and protection of neurons and maintain brain and nervous system function. Under normal circumstances, high levels of S100B protein are mainly found in the cerebrospinal fluid (CSF), but low level of S100B in plasma and brain [7]. After brain injury, the activated microglia can secrete interleukin (IL) such as IL-1β, IL-6, tumor necrosis factor-α, and stimulate the activation and proliferation of glial cells, resulting in a large amount of S100B [8]. In the serum and CSF of patients with major depression, S100B protein has been shown to be increased compared to levels in healthy controls [9, 10], although other studies did not demonstrate this difference in the CSF of MDD patients [11]. A postmortem study found that the density of S100B-immunopositive astrocytes is decreased in the CA1 pyramidal layer of the hippocampus in patients with MDD [12]. In some longitudinal studies, it was reported that higher S100B levels were decreased after treatment with antidepressants [13]. A recent meta-analysis by Schroeter et al. [3] revealed that S100B serum levels were consistently increased in acute major depressive or manic episodes, which was shown to be decreased after treatment with antidepressants, suggesting that S100B may be a biomarker for treatment outcomes in depression [14]. However, Ambrée et al. [15] reported low S100B levels in patients with depression, which predicted nonresponse to venlafaxine. Taken together, these studies suggest that the increased serum S100B levels may be involved in the pathophysiology of MDD and in pharmacological mechanisms of antidepressants.

The hippocampus is an important brain region that can regulate emotions and cognition [16]. Some studies have reported neurochemical changes mainly in the hippocampus in patients with depression [17]. The antidepressant venlafaxine, which is widely used to treat patients with depression, has unique chemical characteristics in inhibiting of 5-hydroxytryptamine (5-HT) and norepinephrine synaptosomal reuptake and increasing brain 5-HT levels more than fluoxetine [18]. To our knowledge, no published studies have directly examined the alteration of S100B mRNA level and protein expression in the hippocampus of depression patients or animal models and the influence of venlafaxine on them. We hypothesized that the anti-depressant activity of venlafaxine is associated with its effects on S100B mRNA level and protein expression in the hippocampus in a rat depression model induced by chronic unpredictable mild stress (CUMS).

## Methods

### Animals

Forty male Sprague-Dawley rats (provided by the Experimental Animal Center of Hebei Province, China), aged 8–10 weeks and weight 240–280 g (SCXK2003-1-003) were housed under standard laboratory conditions and maintained on a 12-h light–dark cycle with free access to food and water. The animals and experimental protocols were approved and supervised by the Institutional Animal Ethics Committee of the Xinxiang Medical University (with the ethical approval number 20090517) and followed the guidelines of the China National Science Academy for the use and care of experimental animals.

### Chronic unpredictable mild stress (CUMS) model

The CUMS procedure was modified based on the methods in previous reports [19]. All rats in the treated groups received a variety of different stimuli from day 2 to day 43: (1) 45° cage tilt (for 24 h); (2) cold temperature swimming (water temperature 4 °C, 30 cm depth, every day for 5 min); (3) shaking (frequency: 1 per second, for 10 min); (4) tail clamp (nipped the rat’s tail side of the body nearly 1/3 with large oval clamp for l min); (5) heat stress (rats placed in narrow-mouthed bottles at 45 °C for 5 min); (6) water deprivation (24 h); (7) food deprivation (24 h); (8) noise stimulation (1500 Hz at 95 dB for l h/day; (9) 120 min limit behavior (the heads of rats were fixed in the end of a cylinder without affecting their breathing); (10) wet bedding (250 ml of water added to the cage with sawdust, 24 h). The rats received one of these ten stimulation styles each day and each stimulus was applied two or three times with a completely random order. However, the same stimulus could not be used consecutively.

### Drugs and drug treatment

Venlafaxine (Southwest Pharmaceutical Co., Ltd., Chengdu, China; Batch number: 080203) were dissolved in normal physiological saline. Rats were treated with venlafaxine at doses of 0, 5 or 10 mg/kg (1 ml/each rat) and were administered by oral gavage once a day for 21 days. The dosages of venlafaxine used in this study were chosen based on previous reports by Chen et al. [20] and Xing et al. [21].

The rats were randomly divided into four groups (n = 10/each group): (a) control group: rats were reared 3–4/cage, and were neither exposed to CUMS nor treated with venlafaxine. (b) active groups: rats were housed individually in the cage and were exposed to CUMS from day 2 to day 43. Then they were administered with different doses of venlafaxine (0, 5 or 10 mg/kg) from day 23 to day 43.

### Behavioral assessment

**Fig. 1:**
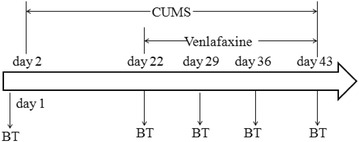
The experimental process. CUMS: the procedure of chronic unpredictable stress from 2nd day to 43rd day; BT: behavioral test on the day 1, day 22, day 29, day 36 and day 43; 5 mg venlafaxine group: the animal received venlafaxine at a dose of 5 mg/day from day 22 to day 43; 10 mg venlafaxine group: the animal received venlafaxine at a dose of 10 mg/day from day 22 to day 43

Behavioral assessments were carried out on day 1, 22, 29, 36 and 43, which were shown in Fig. 1. The tests on day 1 and day 22 were used to examine whether the depression model was successful [19]. The tests on day 29, day 36 and day 43 were to estimate whether the depression-like behaviors was reversed by different doses of venlafaxine at different time points.

### The body weight and the open-field test

The body weight was measured before each the open-field test and at day 1, 22, 29, 36 and 43, respectively to calculate the mean body weight change during the entirety of the experiment. The open-field test included the distance of horizontal motion, the number of vertical motion, which was used to monitor the motor ability and explorative ability of the rats in an unfamiliar environment.

Following [19], rats were individually placed in a small 100 cm × 100 cm × 40 cm Plexiglas cage and allowed 30 s to accommodate to the observation cage. The behavioral parameters of the open-field test were continuously measured for 5 min by three observers at the same time. In all the experiments, the scorers were blinded to animal treatment. Each rat was placed in the center of the open field; the distance of the horizontal motion was recorded by a data acquisition system (software of Smart 2008, Germany). The number of vertical motion (rearing times) was recorded by the trained observers. The vertical motion reflected explorative ability (curiosity) and horizontal motion referred to locomotor activity in an unfamiliar environment. Thus, the decrease of horizontal and vertical motion simulated the hypoactivity and anhedonia symptoms of depression.

### Sucrose preference test

The sucrose preference test was performed as described previously [19, 22, 23], with minor modification. Decreased sucrose consumption was used to mimic the core symptoms of anhedonia in patients with depression. After deprivation of water and food for 12 h (21:00–09:00 h), rats were free to access either of two bottles containing 1% sucrose solution or water. The positions of the two bottles were switched after 30 min. The rats were housed in individual cages. After 1 h, the volumes of consumed sucrose solution and water were recorded. The sucrose preference ratio (SPR) was calculated according to the following equation: SPR = sucrose intake (ml)/sucrose intake (ml) + water intake (ml).

### Immunohistochemistry

Experimental and control animals were sacrificed within 12 h following the last behavior test [23]. All rats were deeply anesthetized with intraperitoneal injection of 0.3 ml/100 g chloral hydrate. The brain was quickly removed, postfixed in 4% paraformaldehyde for 3–4 h, and kept overnight for 12 h. After dehydration and paraffinization, serial 5-μm-thick coronal sections were cut. Sections were incubated for 10 min in 3% hydrogen peroxide to eliminate endogenous peroxidases, and then incubated with rabbit anti-S100B polyclonal antibody (1:100; Santa Cruz Biotechnology, Santa Cruz, CA, USA) overnight at 4 °C. For negative controls, the antibody was replaced with normal goat serum. After removal of the primary antibody, the sections were all washed three times with PBS. Biotinylated goat anti-rabbit antibody (Bo Shi De, Wuhan, China) and streptavidin–biotin complex (Bo Shi De, Wuhan, China) were then each applied for 20 min at 20–37 °C. Color for the peroxidase-linked antibody was developed with diaminobenzidine (DAB) for 5 min at room temperature. Finally, they were photographed under a light microscope [24].

### In situ hybridization

The method of in situ hybridization referred to the research of Bjørnebekk’s et al. [25]. Paraffin sections were fixed to glass slides and washed in water, and treated with 3% hydrogen peroxide at room temperature for 10 min to inactivate endogenous peroxidase. After washing in distilled water three times, tissue sections were placed in 3% newly diluted pepsin with citric acid at room temperature for 30 min. After washing in PBS three times, each time for 5 min and in distilled water three times, sections were fixed in 1% paraformaldehyde/0.1 M PBS (pH 7.4) containing 1/1000 diethylpyrocarbonate at room temperature for 10 min. After washing in distilled water three times, each time for 5 min, the sections were placed in 20 µl pre-hybridization to solution at 38 °C for 2 h, and excess liquid was absorbed without washing. The sections were then placed in 20 µl hybridization solution (S100B oligonucleotide probe to target mRNA sequence was 5′-TTCCA TCAGT ATTCA GGGAG AGAGG GTGAC AAGCA-3′), and the coverslip was placed on the glass slide and hybridized overnight at 38 °C. After removing the coverslip, the sections were washed twice for 1 min with 2× saline-sodium citrate (SSC) buffer, once for 15 min with 0.5× SSC, and once for 15 min with 0.2× SSC at 37 °C. Sections were incubated orderly with blocking buffer at 37 °C for 30 min, biotinylated anti-mouse digoxin (Bo Shi De) for 1 h at 37 °C, streptavidin–biotin complex (Bo Shi De) for 20 min at 37 °C, biotin–peroxidase for 20 min at 37 °C, one drop DAB reagent for 20 min at room temperature. After washing in running water, sections were dehydrated and made transparent. Finally, the coverslip was placed on the glass slide. Sections were visualized with a light microscope. In the negative control, S100B oligonucleotide probes were replaced by 0.1 M PBS (pH 7.4).

### The analysis of the sections in immunohistochemistry and In situ hybridization

The sections were located in 3.3–3.8 mm distance from bregma and used Lecia Image collection and analysis system (DM2000) to take the photographs of immunohistochemistry and in situ hybridization. The photographs were counted by means of Image pro-plus (6.0), which outcomes were adopt by the formula of Mean optical density (MOD) = Integral optical density (IOD)/Area. Four sections were selected to count each rat, and 10 rats were in each group.

### Statistical analysis

To investigate the effects of different dose of venlafaxine on behavior tests and the change in the hippocampal S100B protein expression and mRNA level, we used repeated-measures ANOVA test for interactions of treatment grouping with changes in behavior tests and S100B expression and mRNA level, with baseline and post-treatment values as the dependent measures (within-subject factors). Post hoc comparisons between groups were made using the Bonferronni or Tukey post hoc analysis. In addition, we compared the post-treatment values in the four groups using a univariate analysis of covariance (ANCOVA) with baseline value as covariate. S100B data was analyzed by one-way ANOVA followed by Bonferronni or Tukey post hoc analysis. Data were presented as mean ± SD. Differences at *p* < 0.05 level were considered to be significant. All statistical analyses were performed using SPSS, version 17.0 (Chicago, IL, USA).

## Results

### Body weight

**Fig. 2:**
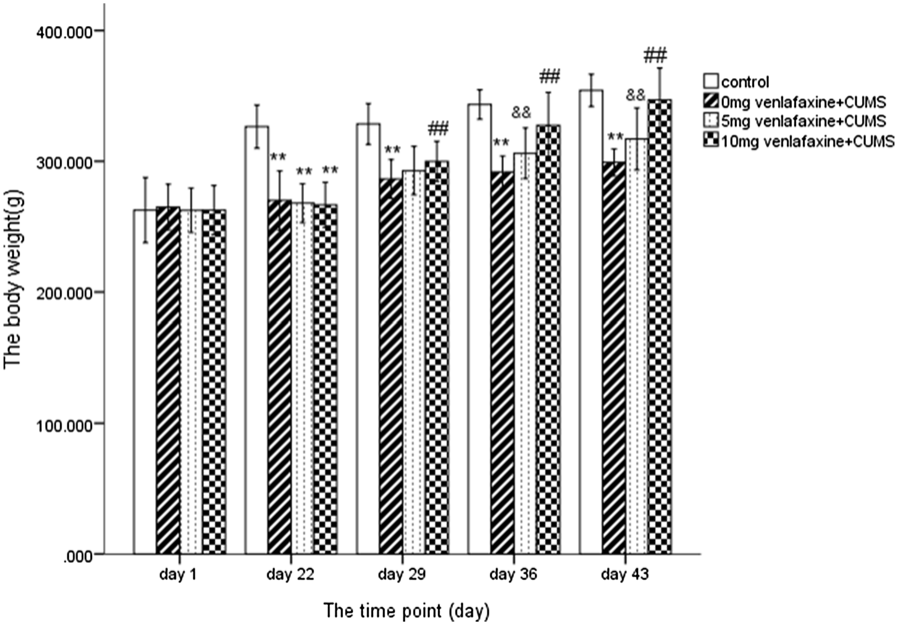
The body weight at different time. The values are expressed as mean ± SD (n = 10/group). Compared with control group *p < 0.05, **p < 0.01. Compared with 0 mg venlafaxine group “^#^ *p* < 0.05, ^##^ *p* < 0.01”. Compared with 10 mg venlafaxine group “^&^ *p* < 0.05, ^&&^ *p* < 0.01”. On day 22, the body weight in control was higher than the other three groups (All *p* < 0.01). On day 29, there was still significant difference between the 0 mg venlafaxine and control group (*p* < 0.01); compared with the 0 mg venlafaxine group, the body weight was higher in the 10 mg venlafaxine. On day 36, there was still significant difference between the 0 mg venlafaxine and 10 mg venlafaxine group (*p* < 0.01). On day 43, the body weight was higher in the 10 mg venlafaxine compared with the 5 mg venlafaxine group

Figure 2 presents the mean body weight on each testing occasion for the different groups. At baseline, there was no statistical difference between the four groups in body weight (F = 0.152, df = 3,36, *p* > 0.05). After 21 days of CUMS, all three treated groups showed significantly lower body weight than control group (0 mg venlafaxine: t = 13.2; 5 mg venlafaxine: t = 10.9; 10 mg venlafaxine: t = 12.794, all *p* < 0.001).

On day 29, there was a significant difference among the four groups (F = 93.2, df = 3,36, *p* < 0.001) and a significant low body weight was found in the three treatment groups compared to control group (*p* < 0.01). On day 36, there was a significant difference among the four groups (F = 88.2, df = 3,36, *p* < 0.001) and high body weight was observed in the 10 mg venlafaxine group than that in the 5 mg venlafaxine group (*p* < 0.001). On day 43, there was a significant difference among the four groups (F = 97.0, df = 3,36, *p* < 0.001) and the body weight in 10 mg venlafaxine group was significantly greater than in the 5 mg venlafaxine group (*p* < 0.001); however, there was no significant difference between the 10 mg venlafaxine group and the control group (*p* > 0.05) (Additional file 1).

### The open-field test

**Fig. 3:**
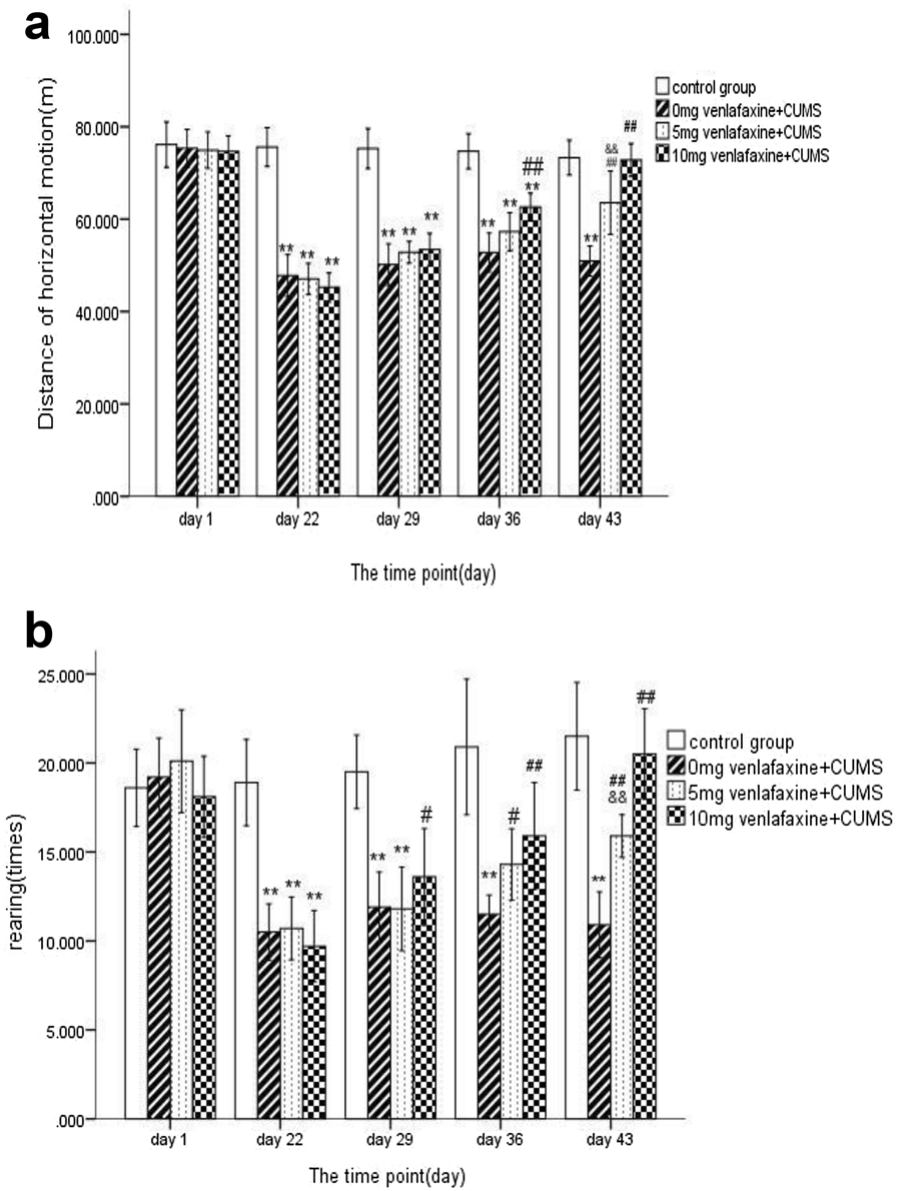
Behaviors of the open-field test at different time. The values are expressed as mean ± SD (n = 10/group). Compared with control group “*p < 0.05, **p < 0.01”. Compared with 0 mg venlafaxine group “^#^ *p* < 0.05, ^##^ *p* < 0.01”. Compared with 10 mg venlafaxine group “^&^ *p* < 0.05, ^&&^ *p* < 0.01”. **a** Distance of horizontal motion; **b** rearing times. On day 22, there were significant differences among the control group and three treated groups in distance of horizontal motion, and rearing times (all *p* < 0.01). On day 29, there was still significant difference between the 0 mg venlafaxine and control group (*p* < 0.01). On day 36, there was still significant difference between the 0 mg venlafaxine and 10 mg venlafaxine group (*p* < 0.01). On day 43, the distance of horizontal motion and number of vertical motion was higher in the 10 mg venlafaxine compared with the 5 mg venlafaxine group (*p* < 0.01)

Figure 3 presents the open-field test results on each testing occasion for the different groups. At baseline, there was no statistical difference between the four groups in the distance of horizontal motion and rearing times among the four groups (F = 0.248, df = 3,36, *p* > 0.05; F = 1.282, df = 3,36, *p* > 0.05). After 21 days of CUMS, all three treated groups showed the decrease in distance of horizontal motion and rearing times compared to the control group (all *p* < 0.001).

On day 29, there was a significant difference in distance of horizontal motion, the rearing times among the four groups (F = 95.4, df = 3,36, *p* < 0.001; F = 25.0, df = 3,36, *p* < 0.001; respectively). Although the behaviors of open-field test have still significant difference between the three treated groups and control group (*p* < 0.01), higher times was found in the number of rearing in venlafaxine group(10 mg) than that in venlafaxine group (0 mg) (*p* < 0.05).

On day 36, there was a significant difference in distance of horizontal motion and rearing times among the four groups (F = 61.8, df = 3,36, *p* < 0.001; F = 21.7, df = 3,36, *p* < 0.001; F = 100.8, df = 3,36, *p* < 0.001, respectively) and the activities of the venlafaxine group (0 mg) were significantly lower than that of venlafaxine groups (5 or 10 mg) (all *p* < 0.05). On day 43, there was a significant difference in distance of horizontal motion, the rearing times among the four groups (F = 51.9, df = 3,36, *p* < 0.001; F = 45.9, df = 3,36, *p* < 0.001) and significant increase in the open-field tests was observed in venlafaxine group (10 mg) compared to venlafaxine group (5 mg) (all *p* < 0.05), but no significant differences in all open-field tests were observed between 10 mg venlafaxine group and control group (all *p* > 0.05) (Additional file 2).

### Sucrose preference test

**Fig. 4:**
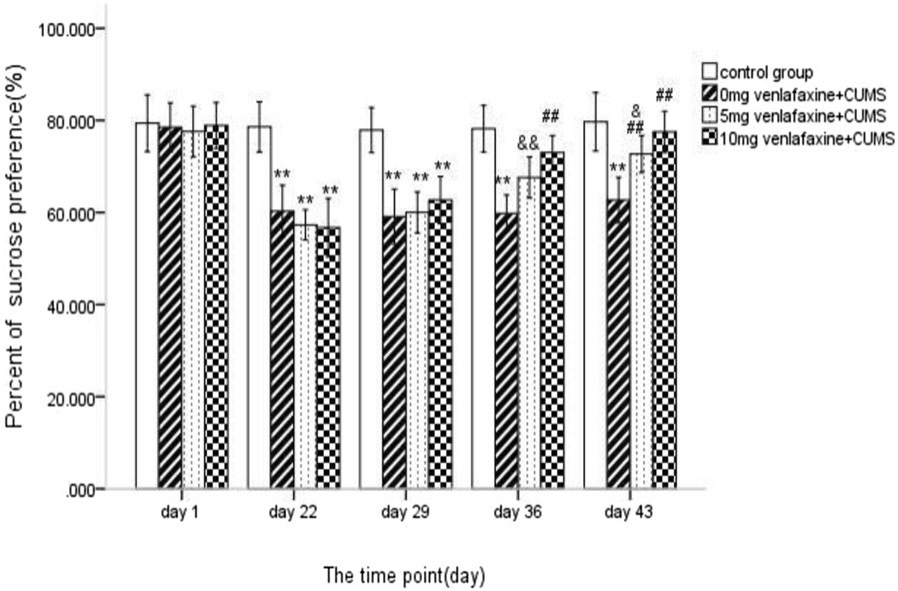
The sucrose preference test at different time. The values are expressed as mean ± SD (n = 10/group). Compared with control group “*p < 0.05, **p < 0.01”. Compared with 0 mg venlafaxine group “^#^ *p* < 0.05, ^##^ *p* < 0.01”. Compared with 10 mg venlafaxine group “^&^ *p* < 0.05, ^&&^ *p* < 0.01”. On day 22, the sucrose preference in control was higher than the other three groups (all *p* < 0.01). On day 29, there was still significant difference between the 0 mg venlafaxine and control group (*p* < 0.01); compared with the 0 mg venlafaxine group, the sucrose preference was higher in the 10 mg venlafaxine (*p* < 0.01). On day 36, there was still significant difference between the 0 mg venlafaxine and 10 mg venlafaxine group (*p* < 0.01). On day 43, the sucrose preference was higher in the 10 mg venlafaxine compared with the 5 mg venlafaxine group (*p* < 0.01)

Figure 4 presents the sucrose preference test results on each testing occasion for the different groups. At baseline, there was no statistical difference in sucrose preference test among the four groups (F = 0.192, df = 3,36, *p* > 0.05). After 21 days of CUMS, all three treated groups showed significantly lower sucrose preference than control group (0 mg venlafaxine t = 13.104, *p* < 0.001; 5 mg venlafaxine t = 9.510, *p* < 0.001; 10 mg venlafaxine t = 9.955, *p* < 0.001).

On day 29, there was a significant difference among the four groups (F = 29.2, df = 3,36, *p* < 0.001) and the control group still showed greater sucrose preference than the treated groups (all *p* < 0.01). On day 36, there was a significant difference among the four groups (F = 32.9, df = 3,36, *p* < 0.001) and both the 0 and 5 mg venlafaxine groups had lower sucrose preference than the 10 mg venlafaxine group (both *p* < 0.001). On day 43, there was a significant difference among the four groups (F = 22.8, df = 3,36, *p* < 0.001) and significant difference was observed between 10 versus 5 mg venlafaxine groups (all *p* < 0.05), but no significant difference was observed between 10 mg venlafaxine group and control group (*p* > 0.05) (Additional file 3).

### Immunohistochemistry and in situ hybridization

**Fig. 5:**
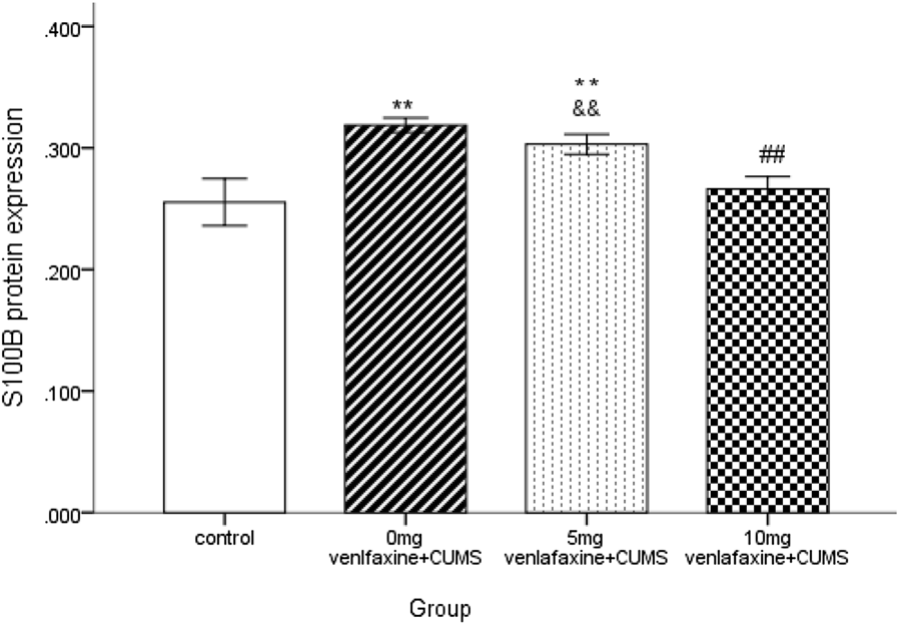
Mean optical density for S100B protein in the hippocampus. The values are expressed as mean ± SD (n = 10/group). Compared with control group “*p < 0.05, **p < 0.01”. Compared with 0 mg venlafaxine group “^#^ *p* < 0.05, ^##^ *p* < 0.01”. Compared with 10 mg venlafaxine group “^&^ *p* < 0.05, ^&&^ *p* < 0.01”. The S100B protein in 0 mg venlafaxine was higher than the other three groups (all *p* < 0.01). There was significant difference between the 10 mg venlafaxine and control group (*p* < 0.01); compared with the 5 mg venlafaxine group, S100B protein was lower in the 10 mg venlafaxine (*p* < 0.01)

**Fig. 6:**
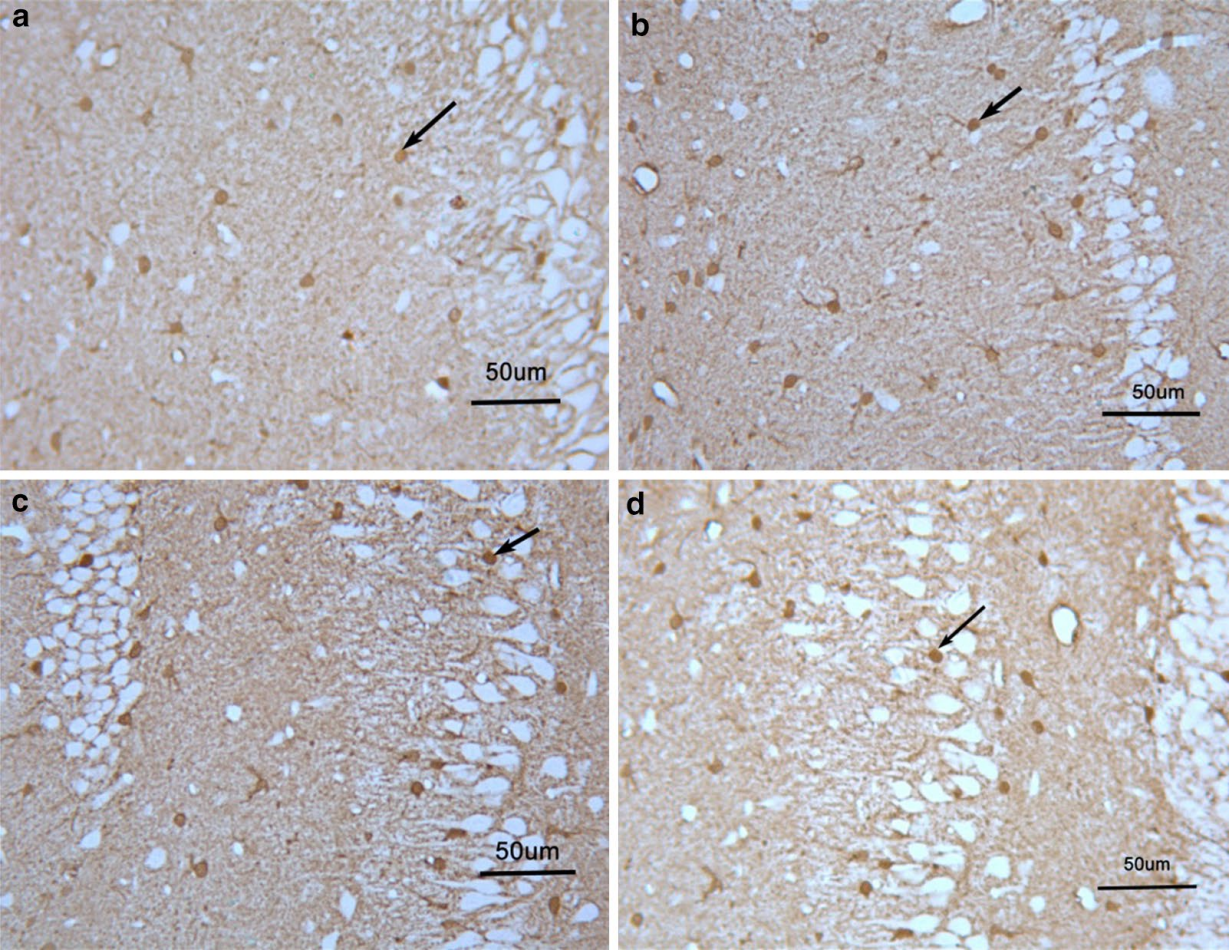
S100B protein pictures in the immunohistochemistry. **a** Control group; **b** 0 mg venlafaxine group; **c** 5 mg venlafaxine group; **d** 10 mg venlafaxine group. The amplification factor of each picture was ×200; the *scale bars of every picture* were 50 μm; →: positive staining

**Fig. 7:**
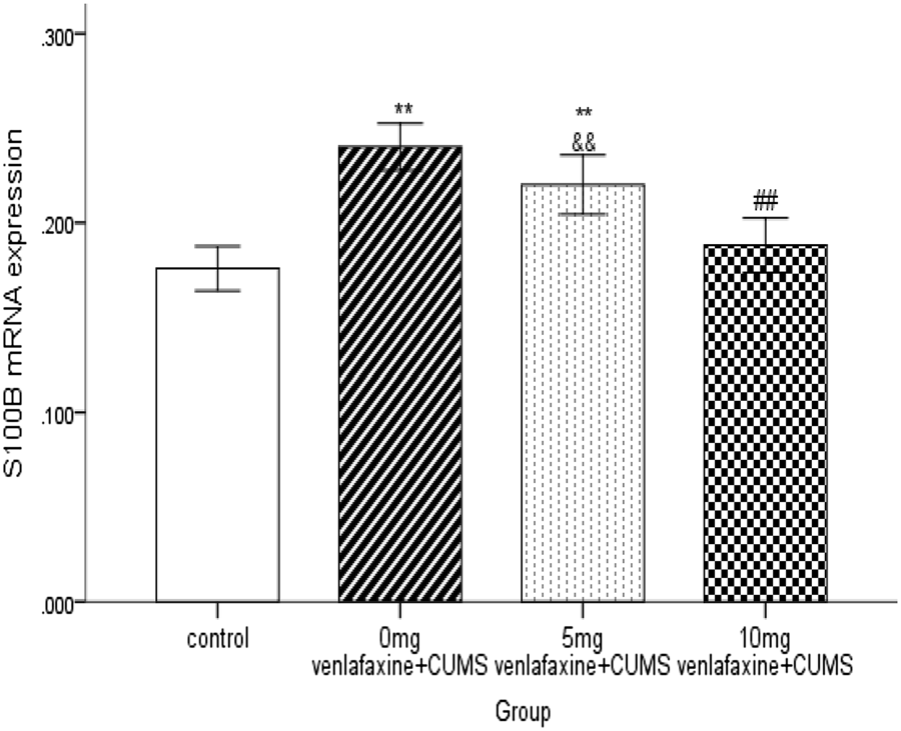
Mean optical density for S100B mRNA in the hippocampus. The values are expressed as mean ± SD (n = 10/group). Compared with control group “*p < 0.05, **p < 0.01”. Compared with 0 mg venlafaxine group “^#^ *p* < 0.05, ^##^ *p* < 0.01”. Compared with 10 mg venlafaxine group “^&^ *p* < 0.05, ^&&^ *p* < 0.01”. The S100B mRNA level in 0 mg venlafaxine was higher than the other three groups (all *p* < 0.01). The significant difference had been found between the 10 mg venlafaxine and 5 m venlafaxine (*p* < 0.01); compared with the 0 mg venlafaxine group, there was no significance in the 10 mg venlafaxine (*p* < 0.01)

**Fig. 8:**
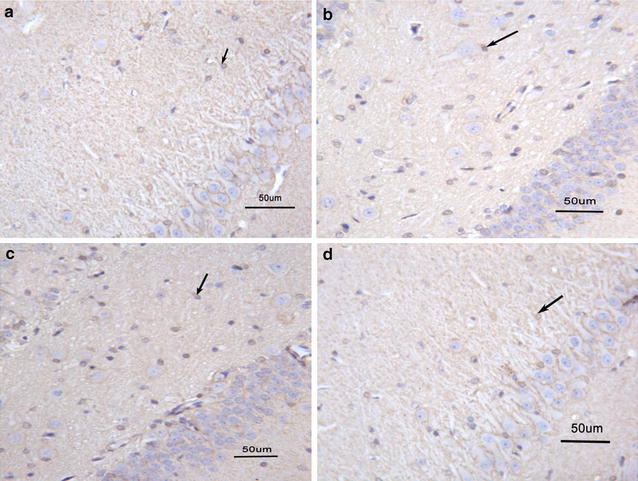
S100B mRNA pictures in situ hybridization. **a** Control group; **b** 0 mg venlafaxine group; **c** 5 mg venlafaxine group; **d** 10 mg venlafaxine group. The amplification factor of each picture was ×200; the scale bars of every picture were 50 μm; →: positive staining

Significant difference in S100B protein expression was observed in the four groups (F = 31.4, df = 3,36, *p* < 0.001). S100B protein expression in saline group was significantly higher than in the other three groups (all *p* < 0.05); however, no significant difference was noted between the control and 10 mg venlafaxine groups (*p* > 0.05). Further, S100B protein expression was significantly greater in the 5 mg venlafaxine group than in the control and 10 mg venlafaxine groups (*p* < 0.001; *p* < 0.01, respectively) (Figs. 5, 6). In addition, significant differences in S100B mRNA expression were observed among the four groups (F = 23.7, df = 3,36, *p* < 0.001), and the control group showed greater S100B mRNA expression than the other three groups (all *p* < 0.05). After 21 days of venlafaxine treatment, S100B mRNA expression was significantly greater in the 5 mg venlafaxine group than in the control and 10 mg venlafaxine groups (*p* < 0.001; *p* < 0.01, respectively); however, no significant difference was noted between the 10 mg venlafaxine and control groups (*p* > 0.05) (Figs. 7, 8) (Additional file 4).

## Discussion

The CUMS-induced behavioral changes may simulate depression-like behaviors in individuals with depression, which is considered as one of the classical models [26]. In our present study, following three weeks of chronic stress, decreases in distance of horizontal motion and the rearing times in an open-field suggested lowered motor ability and explorative ability. Body weight and sucrose consumption, similar to the symptoms of psychomotor inhibition, anhedonia, and appetite decreases in depression [1], were decreased in the treated groups compared to the control group. Taken together, CUMS-induced behavioral changes in our present study paralleled the symptoms of depression in humans.

The present results in this animal model of depression confirm that both 5 and 10 mg venlafaxine administration improved the depression-like behaviors at day 36 and day 43 after 2- and 3-week treatments. Moreover, venlafaxine treatment (10 mg) produced anti-depression effects better than 5 mg venlafaxine treatment at both time points. Interestingly, the depression-like behaviors in rats were reversed fully to normal levels by 21-day treatment of 10 mg venlafaxine, suggesting that high dose of venlafaxine may produce better therapeutic effects than low dose.

A further finding of our present study is that S100B expression and mRNA levels were markedly increased in the hippocampus of the CUMS model of depression suggesting that the depression-like behaviors induced by CUMS might be associated with the elevated S100B mRNA level and protein expression in the hippocampus. Our results were consistent with two previous studies by Gosselin et al. [27], who reported an increase in the intensity of S100b immunoreactivity in prefrontal cortex region, the basolateral amygdala as well as in the hippocampus, and by Ye et al. [28], who reported that CUMS produced increased S100b expression in rat hippocampus.

As mentioned previously, S100B is considered as a potential biomarker of structural brain damage and disease activity [29]. S100B displays neuroprotective or neurodegenerative effects depending on its concentration. At nanomolar concentrations, S100B has been shown to have neurotrophic effects [30], whilst at elevated concentrations (micromolar) is neurotoxic participating in a cascade of events leading to cell death or apoptosis [31]. Numerous studies have shown that S100B is altered in both serum and CSF of patients with mood disorders [3, 32]. Increased S100B levels have been reported in CSF in drug-free mild to moderate depressive patients compared with euthymic patients [33]. Moreover, S100B levels were decreased following successful treatment with antidepressant [34]. The most recent meta-analysis including a very high number of subjects has shown that fluctuations in serum levels of S100B seem to be state markers for major depression [35]. Furthermore, S100B serves as a biochemical predictor of behavioral responses to chronic fluoxetine treatment [36]. Hence, it has been proposed that S100B serum and CSF levels may represent a suitable surrogate marker of glial damage or dysfunction in mood disorders [3, 8]. However, a recent postmortem analysis showed that the numerical density of S100B-immunopositive astrocytes was bilaterally decreased in the CA1 pyramidal layer of the hippocampus in MDD and bipolar disorder (BD) patients compared to controls [12]. The authors assume that reduced glial S100B-immunostaining in the hippocampus of MDD and BD patients is rather caused by an increased release of S100B from glial cells than by reduced cellular S100B expression, because of the observed increase in levels of S100B in the peripheral blood and CSF of MDD and BD patients [8, 10, 14]. Taken together, our finding of increased S100B expression and mRNA levels in the hippocampus of The CUMS model of depression suggests that that S100B overexpression may be a significant marker related to the pathophysiology of the depression.

Interestingly, we found that high dose of venlafaxine at 10 mg not only reversed the CUMS induced behavioral changes in rats, but also reduced increased levels of S100B mRNA level and protein expression. We speculate that venlafaxine may ameliorate the depression-like behaviors by influencing the expression of S100B in the hippocampus.

It was reported that S100B protein stimulated secretion of pro-inflammatory cytokines including IL-1β, IL-6, IL-8 and tumor necrosis factor (TNF)-α through activated glial cells [37, 38], acting as proinflammatory molecule [39]. Therefore, the increased S100B expression in our model of depression may originate from the activated glial cells of the hippocampus and elevate the release of pro-inflammatory cytokines. Recent studies reported that vatairea macrocarpa lectin (VML) caused an enhancement of S100B levels, trigger neuroinflammatory response in mouse hippocampus and exhibited a depressive-like activity [40], suggesting close relationships between the increased S100B and neuroinflammatory markers in the hippocampus and depressive-like behaviors. Some studies have indicated that higher blood levels of pro-inflammatory cytokines TNF-α and IL-6 in drug-free patients with depression [41, 42]. Moreover, the pro-inflammatory cytokines levels were normalized once antidepressant treatment was administered [43]. In addition, it has been demonstrated that the selective serotonin reuptake inhibitors (SSRIs) and serotonin–norepinephrine reuptake inhibitors (SNRIs) exerted anti-inflammatory effects in vivo [44, 45]. Furthermore, Ohgi et al. [46] examined the effects of SSRIs and SNRIs on lipopolysaccharide-induced depression in mice, and found that the two types of antidepressants have anti-inflammatory effects by decreasing TNF-α and increasing IL-10 levels in serum. Also, it has been reported that venlafaxine has an anti-inflammatory effect by the way of suppression on interferon-γ/IL-10 production ratio. The increased hippocampal levels of pro-inflammatory cytokines have been found in the CUMS model of depression, and the improvement of CUMS-induced depression-like behaviors are associated with a reduction of pro-inflammatory cytokines in the rat hippocampus [47, 48]. In addition, the astrocytes have been damaged and their immunoreactivity decrease in the hippocampus of rats exposed to the CUMS [28, 49]. Taken together, we speculate that chronic stress may lead to damage of astrocytes and the hippocampus, causing increases in S100B, which in turn may result in pro-inflammatory cytokines and depression-like symptoms in the CUMS model of depression. After treatment with venlafaxine, the increased expression of S100B mRNA and protein levels was decreased and the release of proinflammatory cytokine declined in the hippocampus, leading to improvements in the depression-like behaviors. However, the mechanisms underlying venlafaxine effects on depression-like behaviors deserve future investigation.

Several limitations of the study should be noted here. First, our study was focused on a single molecular investigation of S100B; however, numerous neurotrophic factors are involved in brain activities. It has been known that there is an interrelationship between different neurotrophins, such as interactions between BDNF and S100B [50]. Second, all the drugs were administered by oral gavage, a very invasive route of administration and source of stress for the animals. Consequently, the oral gavage could be a reason for weight loss observed in the treated rats. A group of naive animals should have been included in the experimental protocol to assess the effect of the stress of the gavage procedure on the evaluated parameters.

In summary, we found that chronic stress led to depression-like behaviors and increased S100B expression and mRNA levels in the hippocampus, suggesting that increased S100B may be relevant to the pathology of depression. Venlafaxine treatment (10 mg) improved these chronic stress induced depression-like behaviors and decreased the elevated S100B levels to the normal range. We speculate that venlafaxine may ameliorate the depression-like behaviors by influencing the expression of S100B in the hippocampus, which may work through its anti-inflammatory effect. However, this is only our speculation. The inter-relationships between increased S100B levels and pro-inflammatory cytokines in the hippocampus and depression deserves further investigation, and especially whether the anti-inflammatory effects of venlafaxine may contribute to its effects on the reduction of S100B level and reversal of the depression-like behaviors warrant further studies.

## Additional files

**Additional file 1.** The data of S100B protein and mRNA.

**Additional file 2.** The data of body weight.

**Additional file 3.** The data of sucrose preference.

**Additional file 4.** The data of open-field test.

## Abbreviations

CUMS: chronic unpredictable mild
MDD: major depression disorder
BD: bipolar disorder
CSF: cerebrospinal fluid
IL: interleukin
TNF: tumor necrosis factor
SSRIs: selective serotonin reuptake inhibitors
SNRIs: serotonin norepinephrine reuptake inhibitors
PBS: phosphate buffer saline
MOD: mean optical density
IOD: integral optical density
VML: vatairea macrocarpa lectins
